# Antimicrobial Activity of Greek Native Essential Oils Against *Escherichia coli* O157:H7 and Antibiotic Resistance Strains Harboring *pNorm* Plasmid, *mecA*, *mcr*-1 and *blaOXA* Genes

**DOI:** 10.3390/antibiotics14080741

**Published:** 2025-07-24

**Authors:** Rafail Fokas, Zoi Anastopoulou, Apostolos Vantarakis

**Affiliations:** Department of Public Health, Medical School, University of Patras, 26504 Patras, Greece; zoi.anastopoulou@ac.upatras.gr

**Keywords:** antimicrobial activity, essential oils, *Escherichia coli*, multidrug-resistant bacteria, minimum inhibitory concentration, minimum bactericidal concentration

## Abstract

Background/Objectives: The rapid emergence of antibiotic-resistant *Escherichia coli* in food and clinical environments necessitates new, clean-label antimicrobials. This study assessed eight Greek native essential oils—oregano, thyme, dittany, rosemary, peppermint, lavender, cistus and helichrysum—for activity against six genetically and phenotypically diverse *E. coli* strains (reference, *pNorm*, *mecA*, *mcr*-1, *blaOXA* and O157:H7). We aimed to identify oils with broad-spectrum efficacy and clarify the chemical constituents responsible. Methods: Disk-diffusion assays measured inhibition zones at dilutions from 50% to 1.56% (*v*/*v*). MIC and MBC values were determined by broth microdilution. GC–MS profiling identified dominant components, and Spearman rank-order correlations (ρ) linked composition to activity. Shapiro–Wilk tests (W = 0.706–0.913, *p* ≤ 0.002) indicated non-normal data, so strain comparisons used Kruskal–Wallis one-way ANOVA with Dunn’s post hoc and Bonferroni correction. Results: Oregano, thyme and dittany oils—rich in carvacrol and thymol—exhibited the strongest activity, with MIC/MBC ≤ 0.0625% (*v*/*v*) against all strains and inhibition zones > 25 mm at 50%. No strain-specific differences were detected (H = 0.30–3.85; *p* = 0.998–0.571; p_adj_ = 1.000). Spearman correlations confirmed that carvacrol and thymol content strongly predicted efficacy (ρ = 0.527–0.881, *p* < 0.001). Oils dominated by non-phenolic terpenes (rosemary, peppermint, lavender, cistus, helichrysum) showed minimal or no activity. Conclusions: Phenolic-rich EOs maintain potent, strain-independent antimicrobial effects—including against multidrug-resistant and O157:H7 strains—via a multi-target mode that overcomes classical resistance. Their low-dose efficacy and GRAS status support their use as clean-label food preservatives or adjuncts to antibiotics or bacteriophages to combat antimicrobial resistance.

## 1. Introduction

The global rise of antibiotic-resistant bacteria constitutes one of the most pressing public health challenges of the 21st century, prompting an urgent demand for alternative antimicrobial strategies. According to recent estimates, bacterial antimicrobial resistance (AMR) directly caused 1.27 million fatalities worldwide in 2019, with another 4.95 million deaths attributable to drug-resistant infections [1]. This escalating crisis is predominantly driven by the misuse and overuse of antimicrobials in human healthcare, animal husbandry and agriculture, accelerating the emergence and dissemination of resistant pathogens [2]. The AMR complicates the treatment of common infections and jeopardizes critical medical procedures such as surgical interventions, organ transplants and chemotherapy, significantly increasing the risk of complications and mortality [3].

Antibiotic overuse and abuse in both clinical and agricultural settings have played a crucial role in the epidemic, allowing multidrug-resistant bacteria to arise and spread quickly [4]. The food supply chain represents a critical transmission pathway for AMR [5]. Resistant bacteria, notably *Escherichia coli*, are frequently detected in animal-derived products, fruits and vegetables, usually resulting from contamination during slaughter, processing, or through the use of untreated animal manure as fertilizer [6]. Additionally, irrigation with dirty water promotes the proliferation of resistant microorganisms. Humans can acquire AMR infections through the consumption of contaminated food or water or via direct contact with animals or contaminated environments [7]. *E. coli* attracted significant scientific attention and public health concern due to its widespread occurrence as a foodborne pathogen and its remarkable capability to acquire and disseminate antibiotic resistance genes [8]. Some *E. coli* strains containing critical resistance determinants such as *pNorm* [9], *mcr*-1 (colistin resistance) [10] and *blaOXA* (beta-lactamase resistance) [11], pose a growing threat because of their ability to withstand multiple antibiotic treatments, complicating patient management and increasing morbidity and mortality. In certain experimental contexts or environmental screenings, the *mecA* gene—typically associated with *Staphylococcus aureus*—has also been detected in *E. coli* or transferred to it under laboratory conditions [12], raising concerns about potential horizontal gene transfer events. Additionally, *E. coli O157:H7* has emerged as a notorious serotype linked to significant foodborne outbreaks across the world, frequently resulting in serious consequences such as hemolytic–uremic syndrome (HUS) [13].

Essential oils (EOs), which are naturally occurring secondary metabolites extracted from plants, have lately received interest as possible antimicrobial agents due to their complex combinations of bioactive chemicals, particularly terpenoids and phenolic compounds [14]. EOs’ antimicrobial properties are attributed to their ability to disrupt bacterial cell membranes, interfere with cellular processes, and inhibit biofilm formation [15], all of which significantly reduce the likelihood of microbial resistance when compared to conventional antibiotics [14,16]. This makes essential oils extremely promising as natural preservatives and medicinal agents, notably in food preservation [17], clinical disinfection and public health efforts. The Mediterranean area, particularly Greece, has a surprisingly rich botanical variety, with plants generating essential oils high in powerful antimicrobial compounds, including carvacrol, thymol, p-cymene and other terpenoids [18,19]. While numerous research have looked at the usefulness of EOs like oregano and thyme, there is still a significant vacuum in the literature addressing the potential of less-studied oils like rosemary (*Rosmarinus officinalis*), sage (*Salvia fruticosa*), cistus (*Cistus ladanifer*) and helichrysum (*Helichrysum italicum*) [20]. Preliminary findings show that these oils have antibacterial properties [21]. Nevertheless, their efficacy against multidrug resistance (MDR) organisms such as *E. coli* is unclear [22]. Recent reviews have reaffirmed the antimicrobial potential of essential oils against MDR foodborne bacteria, emphasizing the importance of species-specific evaluations and the need for standardized experimental approaches to assess their applicability in real-world food systems [23,24]. Further research into their chemical makeup and particular mechanisms of action might reveal crucial information about their potential utility as natural antibacterial agents.

Despite numerous studies evaluating essential oils’ antimicrobial potential against general bacterial populations, little attention has been paid to genetically characterized multidrug-resistant *E. coli* strains carrying significant resistance genes such as *pNorm*’s, *mcr*-1, *mecA* and *blaOXA*. This notable research gap warrants further investigation, particularly on how antibiotic resistance determinants influence bacterial susceptibility to EOs. The present study addresses this crucial research gap by systematically assessing the antimicrobial efficacy of eight essential oils extracted from Greek native plants against *E. coli* O157:H7 and various *E. coli* strains characterized by the presence of these critical antibiotic resistance genes. Employing standardized and robust methodologies, including disk diffusion assays, minimum inhibitory concentration (MIC) and minimum bactericidal concentration (MBC) determination, this investigation aims to establish clear relationships between resistance gene presence and susceptibility patterns to essential oils. Ultimately, the outcomes of this study hold substantial implications for developing targeted and effective natural antimicrobial interventions, contributing decisively not only to mitigating antibiotic resistance that has infiltrated the food chain but also addressing the significant threat posed by *E. coli O157:H7* due to its toxin-producing capabilities. Thus, this holistic and comparative assessment of essential oils’ effectiveness against diverse *E. coli* strains underscores the innovative potential and strategic relevance of natural antimicrobial solutions in safeguarding global food safety standards.

## 2. Results

### 2.1. Disk Diffusion Assay

As shown in Table 1, at the highest tested concentration of 50%, all EOs showed some inhibitory effect against the six *E. coli* bacteria, but the efficacy varied significantly. Oregano (*Origanum vulgare*) had the most impact, continuously producing inhibition zones greater than 35 mm against resistant organisms like *E. coli blaOXA* (40.3 ± 1.5 mm) and *pNorm* (29.7 ± 0.6 mm) at 25% concentration. Dittany (*Origanum dictamnus*) had strong activity, with zones of 28.3 ± 0.6 mm against the lenticule strain and up to 31.7 ± 1.5 mm against *blaOXA*. Thyme (*Thymus vulgaris*) followed closely, especially against *pNorm* and O157:H7, with zones exceeding 25 mm.

As the concentrations were reduced from 25% to 12.5%, these three essential oils (oregano, thyme and dittany) maintained high action against virtually all *E. coli* bacteria, albeit with some reduction in zone size. Thyme exhibited a zone of 26.7 ± 0.6 mm at 12.5% against *E. coli blaOXA*, whereas oregano maintained 20.7 ± 1.2 mm against the *mcr*-1 strain. At concentrations below 6.25%, the range of effective EOs became significantly smaller. Oregano and thyme were the most robust, producing inhibition zones of 9–13 mm at 3.125% and even 1.56% against certain strains. Oregano formed an 8.4 ± 0.5 mm zone at 1.56% against the lenticule strain, while thyme-maintained activity of 7.5 ± 1.3 mm at the same dilution. Other Eos, including peppermint, lavender and rosemary, had lower effectiveness rates, below 12.5%, resulting in zones of less than 8 mm or none at all.

Cistus (*Cistus ladanifer*) and helichrysum (*Helichrysum italicum*) showed little to no action throughout the dilution series. Even at 50% concentration, inhibition zones were either nonexistent or limited (7–8 mm), and in most cases, decreased to 0 mm at concentrations below 6.25%.

Between 24 and 48 h, only minor variations were recorded in the inhibition zone diameters, typically less than ±0.5 mm and never exceeding ±1 mm. No further changes were observed after 48 h, indicating that the maximum antimicrobial effect was reached within the first day of exposure and remained stable thereafter. To contextualize the antimicrobial activity of ΕOs, three conventional antibiotics were included as internal controls in the disk diffusion assays. The inhibition zones and interpretations are shown in Table 2. Results confirmed expected resistance profiles for strains and susceptibility in reference strains, validating assay performance.

### 2.2. MIC and MBC

The MIC and MBC values of the tested EOs against the six *E. coli* strains are listed in Table 3. Among all evaluated EOs, oregano had the strongest antibacterial activity, with MIC values ranging from 0.03125% to 0.125% across all strains. Its bactericidal concentrations were likewise consistently low, with MBCs ranging from 0.0625% to 0.25%, showing a significant bactericidal rather than just bacteriostatic activity. The lowest MIC was found against *E. coli* lenticule and *blaOXA* strains (0.03125%), whereas the lowest MBC (0.0625%) was found against *E. coli* O157:H7. Thyme and dittany were the next most effective EOs, with thyme and dittany having MICs ranging from 0.125% to 0.5% throughout the tested strains. Both oils had consistent MBC values, indicating dependable bactericidal activity. Thyme had an MIC and MBC of 0.125% against *pNorm* and *mecA* strains, which matched oregano’s effectiveness in these specific circumstances. In contrast, peppermint, lavender, rosemary and cistus had significantly higher MIC and MBC values, ranging from 1% to >2%, indicating lesser efficiency and the requirement for greater doses to accomplish microbial control. Lavender and cistus, for instance, had MBCs greater than 2% against various bacteria, indicating inadequate bactericidal activity even at high dosages. Helichrysum was discovered to be mostly inactive, with MIC and MBC values unknown (ND) for five of six strains and only modest activity against *E. coli* O157:H7 (MIC = 2%, MBC > 2%).

### 2.3. Statistical Analysis

Shapiro–Wilk tests (α = 0.05) confirmed significant departures from normality for every response variable (W = 0.706–0.913, *p* ≤ 0.002). Consequently, inter-strain comparisons were performed with the non-parametric Kruskal–Wallis test.

**Figure 1:**
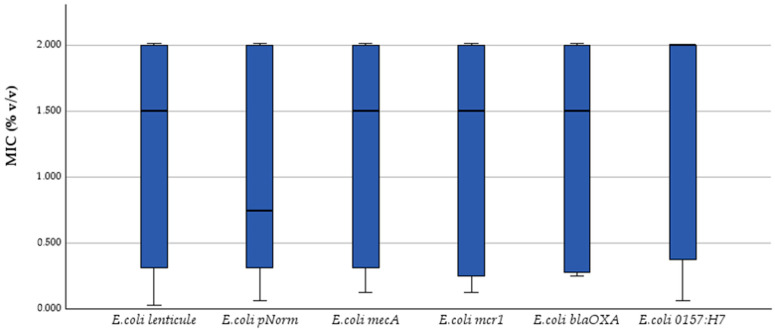
MIC (% *v*/*v*) box-plots for six *E. coli* strains; Kruskal–Wallis *p* = 0.998.

**Figure 2:**
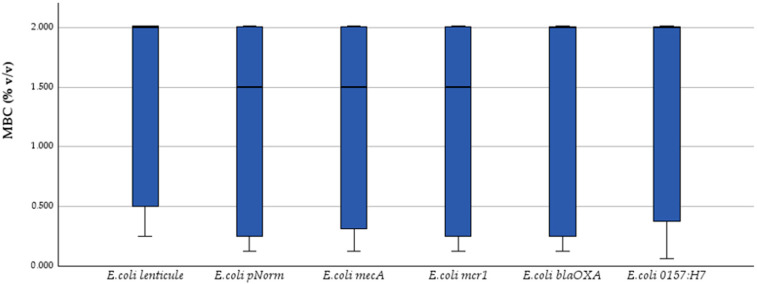
MBC (% *v*/*v*) box-plots for the same strains; Kruskal–Wallis *p* = 0.975.

**Figure 3:**
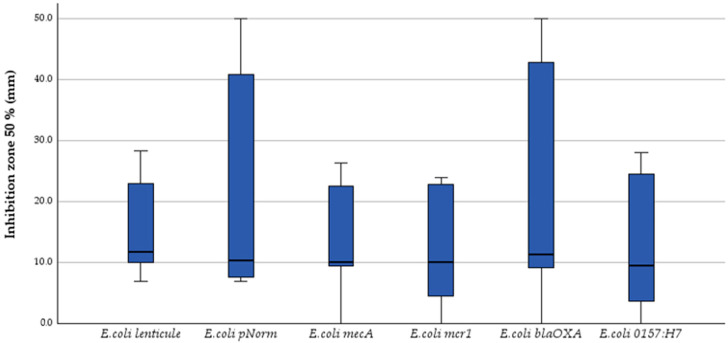
Inhibition-zone diameter at 50% (*v*/*v*) dilution; Kruskal–Wallis *p* = 0.907.

Kruskal–Wallis one-way ANOVA on ranks revealed no significant strain-to-strain variation for any end-point: MIC (H = 0.30, *p* = 0.998) (Figure 1), MBC (H = 0.84, *p* = 0.975) (Figure 2) and inhibition-zone diameters at 50% (Figure 3), 25%, 12.5%, 6.25%, 3.125% and 1.56% *v*/*v* dilutions (H = 1.55, 1.15, 3.38, 3.85, 2.55 and 2.28; *p* = 0.907–0.571). Dunn post hoc contrasts with Bonferroni correction were uniformly non-significant (p_adj_ = 1.000), confirming the absence of strain-specific effects.

Thus, both bacteriostatic (MIC) and bactericidal (MBC) activities, as well as diffusion-based inhibition zones, remained essentially strain-independent, underscoring the functional stability of the tested phenolic-rich essential oils across genetically and serotypically diverse *E. coli* populations.

Spearman rank-order correlations showed that the phenolic monoterpenes carvacrol and thymol are the main determinants of antibacterial potency. Carvacrol content displayed very strong positive correlations with all inhibition-zone diameters (ρ = 0.771–0.881, *p* < 0.001) and very strong negative correlations with both MIC (ρ = –0.858, *p* < 0.001) and MBC (ρ = –0.851, *p* < 0.001). Thymol followed a similar pattern, correlating positively with inhibition zones (ρ = 0.527–0.703, *p* < 0.001) and negatively with MIC (ρ = –0.699) and MBC (ρ = –0.710, both *p* < 0.001). In contrast, terpenes such as α-pinene and 1,8-cineole were inversely related to antimicrobial efficacy: α-pinene was negatively associated with zone diameters (e.g., 50% dilution: ρ = –0.520, *p* < 0.001) and positively associated with MIC (ρ = 0.599, *p* < 0.001) and MBC (ρ = 0.651, *p* < 0.001), while 1,8-cineole correlated positively with MIC (ρ = 0.222, *p* < 0.001) and MBC (ρ = 0.132, *p* = 0.001) and negatively with zone diameters (ρ = –0.342, *p* ≤ 0.017). p-Cymene showed moderate positive correlations with inhibition zones (ρ = 0.437–0.645, *p* ≤ 0.002) but was not significantly linked to MIC/MBC. Menthol, menthone, linalool and linalyl acetate exhibited weak correlations (|ρ| < 0.30, *p* > 0.05), indicating minimal contribution to the overall activity.

## 3. Discussion

This study demonstrated that oregano, thyme and dittany essential oils exhibited the most potent antimicrobial activity against all six *E. coli* strains tested, including the virulent O157:H7 and antibiotic-resistant isolates. These three oils consistently showed the lowest MICs and MBCs across the panel. In contrast, rosemary, peppermint and lavender oils had more moderate effects, requiring higher concentrations to inhibit and kill *E. coli*. *Helichrysum italicum* and *Cistus ladanifer* oils were essentially ineffective against *E. coli*, with no significant growth suppression observed at practical doses.

These findings align with the literature, which consistently ranks oregano and thyme oils among the most active agents against Gram-negative pathogens such as *E. coli* [25]. For example, oregano oil has been reported to achieve MICs as low as 0.05% *w*/*w* against Gram-negative bacteria, markedly lower than those of thyme oil [26]. Although somewhat less potent, thyme oil still shows significant efficacy. Previous studies have found an MIC of ~0.10% *v*/*v* and an MBC of ~0.20% *v*/*v* against *E. coli* O157:H7, whereas in our work, we observed markedly lower thresholds (MIC and MBC = 0.0625%), underscoring the superior potency of the oils tested. Notably, whole thyme oil often outperforms its major constituent alone. Thyme oil has been shown to exert greater antibacterial power against *E. coli* than pure thymol, an effect attributed to synergistic interactions with its minor components [27]. Dittany was similarly potent and its performance is supported by the literature as well. Mitropoulou et al. found *O. dictamnus* EO to inhibit a broad range of bacteria, with an MIC of ~0.27% *v*/*v* for *E. coli*, comparable to oregano’s effectiveness [28]. Peppermint, rosemary and lavender oils showed intermediate efficacy. These oils did inhibit *E. coli* growth, but at higher concentrations (generally in the range of 0.5–2% *v*/*v*) compared to the phenolic-rich oils. For instance, οne previous study reported a peppermint oil MIC of 1% (*v*/*v*) against *E. coli* [29], whereas in our work, the minimum inhibitory and bactericidal concentration for peppermint remained consistently at 2% (*v*/*v*) across all six strains. Accordingly, rosemary EO often exhibits little to no [30] antibacterial activity within the of ~0.2–0.6% *v*/*v*, which is consistently linked to its chemical profile [31]. The literature reports MIC values for lavender oil against *E. coli* that are an order of magnitude higher than oregano. One study found lavender’s MIC against *E. coli* to be as high as 0.5–1%, aligning with our findings [32]. In contrast, several of the remaining EOs exhibited only limited or no inhibitory activity against *E. coli*. Both cistus [33] and helichrysum oils failed to meaningfully suppress growth, a finding that accords with previous reports [34].

The major bioactive constituents—chiefly carvacrol and thymol—exert a multifaceted antimicrobial effect, primarily by disrupting membrane permeability and core cellular functions [35,36]. Their hydrophobic character enables them to insert into the lipid bilayer, causing leakage of intracellular contents, collapse of the electrochemical gradient and, ultimately, cell lysis [37,38]. This observation agrees with reports that oregano oil exhibits potent bactericidal activity against a range of MDR pathogens [39], including *E. coli* [40], precisely because it inflicts extensive damage on bacterial membranes and associated proteins. Overall, EO efficacy was unaffected by the presence of resistance genes, underscoring their potential value as alternative antimicrobials when conventional antibiotics fail. Recent mechanistic hypotheses suggest that EO constituents may also disrupt quorum sensing, efflux pump activity and metabolic enzyme function in MDR bacteria [41]—complementing their primary membrane-targeting effects and explaining their sustained efficacy even in resistant strains [14,15,16].

The chemical composition of the EOs proved decisive for their antimicrobial performance. It is worth noting that the variability observed in previous studies on EO antimicrobial efficacy can be attributed to differences in plant chemotypes, geographic and seasonal origin, extraction techniques and testing methodologies. These inconsistencies often limit comparability across studies. In contrast, the present work utilized standardized EO concentrations, triplicate replicates and chemically profiled oils, while assessing activity against well-characterized *E. coli* strains with defined resistance genes. This approach enhances both the reproducibility and the translational relevance of our findings. The particularly high levels of phenolic monoterpenes, in oregano, dittany and thyme oils largely explain their superiority. Gas Chromatography–Mass Spectrometry (GC–MS) profiling showed that our oregano oil contained carvacrol as the dominant constituent (>80–90%), whereas thyme oil was rich in thymol and p-cymene (together ~ 60%). Dittany oil likewise displayed a substantial carvacrol fraction (≈55%) alongside related monoterpenes such as p-cymene (~14%). These phenolic compounds are well documented for their strong antibacterial effects [42]. In our dataset, Spearman analysis revealed a clear positive correlation between an oil’s carvacrol/thymol content and its antimicrobial efficacy, in line with earlier reports. Oregano oils enriched in carvacrol consistently show superior bactericidal action [43], while studies on dittany confirm that carvacrol is the principal contributor to its bioactivity [44]. Beyond the intrinsic strength of the main phenolics, the complex EO matrix can generate synergistic or additive effects [45,46].

The less effective oils (rosemary, peppermint, lavender) have chemical profiles dominated by terpene hydrocarbons or esters rather than phenolic compounds. For example, peppermint oils often contain menthol and menthone as key components [47]; these have moderate antimicrobial effects but again are less potent than carvacrol. Lavender oil’s major constituents include linalool and linalyl acetate [48], while rosemary oil is rich in camphor, α-pinene and 1,8-cineole (eucalyptol) [49]. These compounds, while not inert, generally exhibit milder antimicrobial activity than phenolics.

The inactivity of cistus and helichrysum oils can likewise be attributed to their composition. Although some cistus chemotypes have been reported to inhibit Gram-negative pathogens, including *Salmonella* and *E. coli*, those preparations contained high levels of phenolic constituents [50], whereas our chemotype was dominated by less active terpenes, hence its modest effect. Similarly, helichrysum oil consists mainly of non-phenolic terpenes (e.g., neryl acetate, α-pinene, γ-curcumene) [51], to which *E. coli* is essentially tolerant. A commercial *H. italicum* oil showed noteworthy activity against Gram-positive bacteria (*Staphylococcus aureus*, *Bacillus* spp., MIC 5–10 µg mL^−1^) but none against *E. coli* [34]. The most likely explanation is that its major constituents either cannot penetrate the Gram-negative outer membrane effectively or lack sufficient intrinsic potency. In general, oils lacking appreciable phenolic terpenoids failed to act strongly against this Gram-negative pathogen [52], consistent with the broader observation that Gram-negative bacteria are relatively refractory to many plant extracts because the lipopolysaccharide (LPS) barrier restricts entry [53].

An important outcome of this study is that the essential oils retained full efficacy across all tested resistance genotypes and pathogenic backgrounds. The oils’ antimicrobial activity was essentially unaffected by the presence of antibiotic resistance genes such as *pNorm*, *mecA*, *mcr*-1 or *blaOXA*, and was equally potent against the enterohemorrhagic *E. coli* O157:H7 strain. This breadth is notable since the agri-food continuum is now recognized as a significant reservoir and transmission route for antibiotic-resistant bacteria [6], and recent O157:H7 outbreaks [54] highlight the importance of new control methods.

Resistance genes typically confer protection against specific antibiotics, yet these mechanisms cannot withstand essential oils’ simultaneous disruption of membranes, proteins and DNA [55]. For instance, cinnamon and thyme oils have shown strong inhibitory activity against *E. coli* isolates harboring *mcr*-1, comparable to their activity on colistin-sensitive strains [56]. Additionally, certain thyme oils have been observed to act synergistically with colistin on *mcr*-1-positive *E. coli*, restoring colistin susceptibility through membrane permeabilization [57]. Similarly, the presence of the *blaOXA* β-lactamase gene did not diminish the oils’ bactericidal action. This is expected, as enzymes like OXA-type carbapenemases target β-lactam drug molecules [58] but have no effect on the complex mixture of phytochemicals in an essential oil. In essence, there is no cross-resistance between conventional antibiotics and essential oils because their modes of action are entirely different [59]. Our data exemplify this; even a strain artificially equipped with *mecA* was not better protected against the oils. Although the *mecA* gene is conventionally associated with methicillin resistance in *S. aureus* [60], its sporadic detection in environmental Gram-negative bacteria has been reported, suggesting possible horizontal gene transfer events [12,61]. However, to date, there is no conclusive evidence of stable integration or functional expression of *mecA* in clinical *E. coli* isolates [62]. In the present study, the inclusion of an *mecA*-positive *E. coli* strain—acquired for research purposes—was intended to model a hypothetical but epidemiologically relevant scenario of resistance gene dissemination. Our goal was not to demonstrate *mecA* activity in *E. coli*, but to evaluate whether essential oils retain their antimicrobial effects across genetically diverse resistance backgrounds. Indeed, even when bacteria are serially exposed to sublethal doses of EOs, they rarely develop stable resistance, unlike with antibiotics [63]. It is also noteworthy that the highly pathogenic *E. coli* O157:H7 strain was as sensitive to the oils as non-O157 strains. Yet, our results indicate that oregano, thyme and dittany oils were uniformly lethal to O157:H7 at low concentrations, just as they were to the other isolates. Numerous works have shown oregano and thyme EOs to be highly effective against *E. coli* O157:H7 in vitro and even in food matrices [64].

The effective suppression of *E. coli*, including the O157:H7 serotype, by natural EOs suggests they can serve as clean-label preservatives [65], reducing reliance on synthetic chemicals. Consumer demand is already shifting toward “cleaner” ingredient lists free of nitrites or sorbates [66], creating a niche for plant-derived antimicrobials [67]. Oregano and thyme oils, rich in potent phenolic compounds and sourced from edible herbs, are generally recognized as safe (GRAS/FDA approved) at low doses and have been piloted as antimicrobial food additives [17]. For instance, edible coatings enriched with thyme or oregano oil can be applied to fresh meat [68], fresh fruits [69], cheese or produce [70], forming a thin film that slows pathogen growth. EOs can also be incorporated into active-packaging systems—antimicrobial sachets or films that slowly volatilize the oil and provide continuous protection during storage [71]. However, despite their promising antimicrobial properties, the direct application of essential oils in food systems is not without limitations. Factors such as volatility, oxidation, interactions with food components (e.g., proteins and fats) and strong sensory impact (e.g., flavor, aroma) may affect their stability and consumer acceptability [72]. These aspects must be carefully addressed in future formulation strategies to enhance their practical applicability.

These oils’ ability to suppress both pathogenic and resistance-encoded bacteria at low MIC/MBC thresholds (≤0.0625% *v*/*v*) distinguishes them as very versatile antimicrobials. At such minimal concentrations, they can be incorporated into foods, contact surfaces or active-packaging systems with negligible sensory or toxicological impact—advantages seldom matched by conventional chemical preservatives. Crucially, their multi-target mode of action bypasses specific resistance mechanisms, creating a robust barrier against the spread of resistant organisms throughout the food chain. Moreover, our work underpins the potential for these oils to be used alone or in combination with existing—now less effective—antibiotics to restore or enhance their efficacy. Emerging strategies even explore synergistic applications of essential oils with bacteriophages [72], leveraging phages’ targeted lytic activity [73] together with oils’ broad-spectrum membrane disruption [74]. In an era when the agri-food sector urgently needs clean-label, effective alternatives to synthetic sanitizers and failing antibiotics, these essential oils offer a timely, innovative solution for strengthening food safety systems and combating antimicrobial resistance.

## 4. Materials and Methods

### 4.1. Essential Oils

This study used eight EOs (Table 4) sourced from local Greek aromatic plants. All oils were received from Vessel Essential Oils, a recognized distillation and processing firm that specializes in organically and bio-grown plant materials. The plant material utilized in EO manufacturing was sourced from a variety of locales around Greece, including mountainous and coastal areas, each with its own microclimate, which contributes to the range and richness of essential oil composition. To retain volatile components, each essential oil was extracted using steam distillation shortly after plant gathering. To retain bioactive components, oils were kept in amber glass vials at 4 °C after extraction.

### 4.2. GC–MS Chemical Profiling of Essential Oils

The chemical composition of each EO was determined as part of standard internal quality control procedure. Analyses were performed on an HP-5MS capillary column (30 m × 0.25 mm, 0.25 μm) with helium as the carrier gas (1 mL/min) and electron impact ionization at 70 eV [GC–MS system: 6890 Network GC System coupled to a 5975B MSD, Agilent Technologies, Santa Clara, CA, USA]. Retention indices were calculated using a C8–C20 alkane standard series, and compound identification was based on Wiley/NIST05 libraries. Results are expressed as relative area percentages and presented in Table 5.

### 4.3. Bacterial Strains

Six distinct *Escherichia coli* strains were employed in this study to assess the antimicrobial efficacy of the selected essential oils. These contained one standard reference strain, one surrogate non-toxigenic O157:H7 strain, and four genetically defined MDR strains, each with its own antibiotic resistance gene. The standard non-resistant *E. coli* strain was acquired as a certified reference material in the form of LENTICULE^®^ discs [CRM09001M, lot no. BCCH4935, Supelco, Sigma-Aldrich, Buchs, Switzerland], which included freeze-dried *E. coli* NCTC 9001 (batch SQNL).

The four MRD strains were chosen for their known resistance genotypes and were all laboratory-constructed reference strains provided by Dr. Uli Klümper (Environmental Scences, Technical University of Dresden, Institute of Hydrobiology, Dresden, Germany) as part of the NORMAN project [75]. These strains are commonly used as standardized ARG-positive controls in studies quantifying antimicrobial resistance. The *pNorm* strain (*E. coli* TOP10 harboring the *pNORM*1 plasmid) has a collection of synthetic resistance genes—*intI1*, *blaTEM*, *vanA*, *sul1*, *qnrS1*, *ctx-m-32* and *16S rRNA*—built on a pEX-A2 plasmid backbone and selected with ampicillin. Eurofins Genomics [Eurofins Genomics, Ebersberg, Germany] developed the plasmid, which is commonly utilized in resistance quantification investigations. An *mecA*-positive *E. coli* strain was added due to the uncommon but proven horizontal transfer of the methicillin resistance gene from *S. aureus* to Enterobacterales. Gene presence was verified using PCR. The *mcr*-1-positive *E. coli* strain linked with plasmid-mediated colistin resistance was obtained from a reputable source. Before performing antimicrobial testing, the *mcr*-1 gene was molecularly confirmed. A *blaOXA*-positive *E. coli* with an OXA-type β-lactamase was revived from a cryopreserved stock and tested using molecular tests for inclusion in the study.

The *E. coli* O157:H7 strain, although representing a clinically relevant Shiga toxin-producing serotype, was employed here in its non-toxigenic surrogate form due to biosafety considerations. It was included because to its frequent involvement in foodborne outbreaks and major importance in food safety evaluations. The strain was purchased as a lyophilized tablet with catalog number LGC-ATCC-43895 and batch number 17F13-LGC-ATCC-43895 [LGC Standards, Middlesex, UK; Easy-Tab™ format] and revived using normal microbiological techniques.

Strains were grown in Tryptic Soy Broth [TSB, Oxoid, Basingstoke, Hampshire, UK] and incubated at 37 °C for 18–24 h to achieve logarithmic growth. Bacterial suspensions were adjusted to an optical density of OD625 = 0.08–0.13, comparable to 0.5 McFarland standard (~10^8^ CFU/mL), and utilized immediately for antimicrobial susceptibility testing after strain viability and integrity were verified.

### 4.4. Disk Diffusion Method

The antibacterial activity of the EOs was evaluated using the disk diffusion method, as described by EUCAST [76]. Suspensions of the *E. coli* strains were prepared to contain approximately 10^8^ colony-forming units per milliliter (CFU/mL) and spread evenly onto Mueller–Hinton agar plates. Each EO was diluted in dimethyl sulfoxide (DMSO) to yield six final concentrations (% *v*/*v*): 50%, 25%, 12.5%, 6.25%, 3.125% and 1.56%. Sterile paper disks (6 mm diameter) were impregnated with 10 μL of each EO concentration and placed aseptically onto the inoculated agar plates. Pure DMSO served as the negative control. Antibiotic disks, including ciprofloxacin (5 μg), gentamicin (10 μg) and cefotaxime (30 μg), were used as positive controls, where appropriate. Additionally, ampicillin (10 μg), erythromycin (15 μg) and oxytetracycline (30 μg) were included to assess phenotypic resistance patterns across all *E. coli* strains. Interpretations were based on EUCAST v2025 breakpoints for ampicillin; no official breakpoints exist for erythromycin and oxytetracycline in Enterobacterales, and therefore, results for these agents are reported without formal classification. Plates were incubated at 37 °C for 24, 48 and 72 h, and the diameters of inhibition zones (in mm) around each disk were measured, including the disk diameter. Each test was performed in triplicate to ensure reliability and reproducibility of the results.

### 4.5. MIC and MBC Determination

The minimum inhibitory concentration (MIC) and minimum bactericidal concentration (MBC) of each EO were determined using the standard EUCAST [76] broth microdilution method, adapted for EOs. EOs were diluted in DMSO and then serially two-fold diluted directly in Mueller–Hinton broth (MHB) to achieve concentrations ranging from 2% to 0.00049% (*v*/*v*), through twelve sequential dilutions. Bacterial suspensions adjusted to 0.5 McFarland standard (~10^8^ CFU/mL) were diluted to achieve a final inoculum of approximately 5 × 10^5^ CFU/mL in each well. Microtiter plates were incubated at 37 °C for 24 h. The MIC was defined as the lowest concentration that showed no visible bacterial growth. For MBC determination, 100 μL from wells without visible growth were spread onto Mueller–Hinton agar plates and incubated at 37 °C for a further 24 h. The MBC was defined as the lowest concentration resulting in no colony formation. All tests were conducted in triplicate.

### 4.6. Statistical Analysis

All antimicrobial experiments, including disk diffusion, MIC and MBC assays, were performed in triplicate to ensure reproducibility. Data were initially assessed for normality using the Kolmogorov–Smirnov test and visual inspection of Q–Q plots. Given the non-parametric nature of the data, appropriate non-parametric tests were applied. The Kruskal–Wallis test was used to evaluate potential differences in antimicrobial activity across the six *E. coli* strains for each EO. When required, pairwise comparisons were conducted using Dunn’s post hoc test with Bonferroni correction. Additionally, Spearman’s rank correlation analysis was employed to explore potential associations between the dominant chemical components of the EOs and their antimicrobial activity. All statistical analyses were carried out using IBM SPSS Statistics, version 29.0. The level of statistical significance was set at *p* < 0.05.

## 5. Conclusions

This comprehensive evaluation of eight Greek native essential oils against six genetically and phenotypically diverse *E. coli* strains, including multidrug-resistant isolates (*pNorm*, *mecA*, *mcr*-1, *blaOXA*) and the highly virulent O157:H7, demonstrates that carvacrol- and thymol-rich oils (oregano, thyme, dittany) achieve consistent bacteriostatic and bactericidal effects at remarkably low MIC/MBC thresholds (≤0.0625% *v*/*v*). This strain-independent potency reflects a multi-target mode of action—membrane disruption, protein denaturation and nucleic acid damage—that overcomes classical resistance mechanisms and affords no cross-protection from antibiotic resistance genes. By contrast, oils dominated by non-phenolic terpenes (rosemary, peppermint, lavender, cistus, helichrysum) required far higher doses or proved inactive. These findings not only validate phenolic-rich essential oils as robust, clean-label antimicrobial agents for food and surface applications but also lay the groundwork for innovative synergies with conventional antibiotics or bacteriophages, offering a timely strategy to reinforce food safety barriers and combat antimicrobial resistance.

## Figures and Tables

**Table 1:** Inhibition zones of selected essential oils against *E. coli* strains, determined by disk diffusion assay.

**Essential Oil**	***E. coli* (lenticule)**											
	**24 h**						**48 h and 72 h**					
	**50%**	**25%**	**12.5%**	**6.25%**	**3.125%**	**1.56%**	**50%**	**25%**	**12.5%**	**6.25%**	**3.125%**	**1.56%**
**Peppermint**	10.0 ± 0.0	9.3 ± 1.5	7.5 ± 0.5	7.0 ± 0.0	6.5 ± 0.5	0.0 ± 0.0	9.6 ± 0.4	9.0 ± 0.0	7.7 ± 0.3	7.2 ± 0.2	6.3 ± 0.3	6.1 ± 0.6
**Oregano**	**22.1** ** ± ** **0.9**	**19.4** ** ± ** **0.5**	**18.4 ** **± 0** **.5**	**11.2** ** ± ** **1.1**	**9.2** ** ± ** **1.3**	**8.4** ** ± ** **0.5**	**21.4** ** ± ** **0.5**	**19.1** ** ± ** **0.9**	**17.5** ** ± ** **1.7**	**11.1** ** ± ** **1.0**	**9.1** ** ± ** **1.2**	**8.2** ** ± ** **0.8**
**Dittany**	**28.3 ** **± 0.6**	**24.0** ** ± ** **1.7**	**19.0** ** ± ** **0.0**	**12.7** ** ± ** **1.2**	**9.7** ** ± ** **1.5**	**9.3** ** ± ** **0.6**	**27.7** ** ± 0.6**	**21.7** ** ± ** **2.9**	**15.0** ** ± ** **2.0**	**12.0** ** ± ** **1.7**	**10.0** ** ± ** **1.0**	**9.7** ** ± ** **1.5**
**Rosemary**	11.0 ± 1.0	11.7 ± 2.1	8.7 ± 0.6	6.3 ± 0.6	7.3 ± 0.6	0.0 ± 0.0	10.7 ± 0.6	10.0 ± 1.0	9.3 ± 0.6	7.3 ± 0.4	7.0 ± 0.6	0.0 ± 0.0
**Thyme**	**24.0** ** ± ** **3.6**	**17.7** ** ± ** **1.2**	**15.0** ** ± 0.6**	**11.0** ** ± 0.6**	**8.3** ** ± ** **0.6**	**7.5** ** ± ** **1.3**	**23.3** ** ± ** **2.9**	**15.7** ** ± ** **1.2**	**14.3** ** ± ** **2.5**	**11.7** ** ± ** **2.3**	**8.0** ** ± ** **0.0**	**7.5** ** ± ** **1.3**
**Lavender**	10.0 ± 0.0	10.0 ± 1.7	7.3 ± 0.6	7.0 ± 0.6	0.0 ± 0.0	0.0 ± 0.0	9.3 ± 0.6	9.0 ± 1.0	7.7 ± 0.6	7.0 ± 0.0	0.0 ± 0.0	0.0 ± 0.0
**Cistus**	12.7 ± 0.6	11.5 ± 2.1	0.0 ± 0.0	0.0 ± 0.0	0.0 ± 0.0	0.0 ± 0.0	10.7 ± 2.9	0.0 ± 0.0	0.0 ± 0.0	0.0 ± 0.0	0.0 ± 0.0	0.0 ± 0.0
**Helichrysum**	7.0 ± 0.0	6.5 ± 0.7	6.5 ± 0.0	0.0 ± 0.0	0.0 ± 0.0	0.0 ± 0.0	7.0 ± 0.0	0.0 ± 0.0	0.0 ± 0.0	0.0 ± 0.0	0.0 ± 0.0	0.0 ± 0.0
**Essential Oil**	***E. coli (pNorm)***											
	**24 h**						**48 h and 72 h**					
	**50%**	**25%**	**12.5%**	**6.25%**	**3.125%**	**1.56%**	**50%**	**25%**	**12.5%**	**6.25%**	**3.125%**	**1.56%**
**Peppermint**	8.3 ± 0.6	7.3 ± 0.6	7.0 ± 0.0	0.0 ± 0.0	0.0 ± 0.0	0.0 ± 0.0	8 ±	7.3 ± 0.6	7 ± 0.0	0.0 ± 0.0	0.0 ± 0.0	0.0 ± 0.0
**Oregano**	**OMZ**	**29.7** ** ± ** **0.6**	**26.7** ** ± ** **2.9**	**17.7** ** ± ** **0.6**	**14** ** ± ** **1.0**	**10.7** ** ± ** **0.6**	**OMZ**	**29.3** ** ± ** **1.2**	**24.3** ** ± ** **0.6**	**17** ** ± ** **0.0**	**12.7** ** ± ** **0.6**	**10** ** ± ** **0.0**
**Dittany**	**31.7** ** ± ** **2.9**	**28.3** ** ± ** **1.5**	**14.3** ** ± ** **0.6**	**11.3** ** ± ** **0.6**	**10** ** ± ** **0.0**	**8.3** ** ± ** **0.6**	**29.3** ** ± ** **1.2**	**27.3** ** ± ** **2.5**	**13.7** ** ± ** **0.6**	**11.3** ** ± ** **0.6**	**9.7** ** ± ** **0.6**	**8.7** ** ± ** **0.6**
**Rosemary**	11 ± 1.7	8 ± 0.6	7.3 ± 0.6	7.0 ± 0.0	0.0 ± 0.0	0.0 ± 0.0	11 ± 1.7	8.3 ± 0.6	7 ± 0.6	7 ± 0.0	0.0 ± 0.0	0.0 ± 0.0
**Thyme**	**OMZ**	**36.7** ** ± ** **4.2**	**17.7** ** ± ** **2.5**	**12** ** ± ** **0.0**	**10** ** ± ** **0.0**	**8** ** ± ** **0.0**	**OMZ**	**36.7** ** ± ** **4.2**	**17.7** ** ± ** **2.5**	**12** ** ± ** **0.0**	**10** ** ± ** **0.0**	**8** ** ± ** **0.0**
**Lavender**	9.7 ± 1.2	8 ± 0.0	7.0 ± 0.0	7.0 ± 0.0	7.0 ± 0.0	0.0 ± 0.0	9.7 ± 1.2	8 ± 0.0	7 ± 0.0	7 ± 0.0	7 ± 0.0	0.0 ± 0.0
**Cistus**	7 ± 0.0	7 ± 0.0	0.0 ± 0.0	0.0 ± 0.0	0.0 ± 0.0	0.0 ± 0.0	7.0 ± 0.0	7.0 ± 0.0	0.0 ± 0.0	0.0 ± 0.0	0.0 ± 0.0	0.0 ± 0.0
**Helichrysum**	7 ± 0.0	7 ± 0.0	0.0 ± 0.0	0.0 ± 0.0	0.0 ± 0.0	0.0 ± 0.0	7.0 ± 0.0	7.0 ± 0.0	0.0 ± 0.0	0.0 ± 0.0	0.0 ± 0.0	0.0 ± 0.0
**Essential Oil**	***E. coli* (*mecA*)**											
	**24 h**						**48 h and 72 h**					
	**50%**	**25%**	**12.5%**	**6.25%**	**3.125%**	**1.56%**	**50%**	**25%**	**12.5%**	**6.25%**	**3.125%**	**1.56%**
**Peppermint**	10 ± 0.0	10.7 ± 1.2	9.3 ± 1.2	7.7 ± 1.2	7.7 ± 1.5	7.5 ± 0.0	10 ± 0.0	10.7 ± 1.2	8.3 ± 0.6	7.7 ± 1.2	8 ± 0.0	7 ± 0.0
**Oregano**	**26.3** ** ± ** **3.2**	**23** ** ± ** **1.0**	**20** ** ± ** **0.0**	**15.7** ** ± ** **2.1**	**12.3** ** ± ** **1.5**	**10** ** ± ** **0.0**	**26.7** ** ± ** **2.9**	**23** ** ± ** **2.0**	**20.7** ** ± ** **3.1**	**15** ** ± ** **1.0**	**12** ** ± ** **0.0**	**10** ** ± ** **0.0**
**Dittany**	**21.7** ** ± ** **1.5**	**18.3** ** ± ** **1.5**	**14.7** ** ± ** **0.6**	**11.3** ** ± ** **1.2**	**8.7** ** ± ** **0.6**	**7** ** ± ** **0.0**	**21** ** ± ** **1.7**	**17.3** ** ± ** **2.5**	**13.3** ** ± ** **0.6**	**10.3** ** ± ** **0.6**	**8** ** ± ** **1.0**	**7** ** ± ** **0.0**
**Rosemary**	9 ± 1.0	8.3 ± 0.6	7.7 ± 0.6	7 ± 0.0	0.0 ± 0.0	0.0 ± 0.0	8 ± 0.6	8.3 ± 1.5	7.3 ± 0.6	0.0 ± 0.0	0.0 ± 0.0	0.0 ± 0.0
**Thyme**	**23.3** ** ± ** **2.9**	**19.3** ** ± ** **1.2**	**13.7** ** ± ** **1.2**	**11.3** ** ± ** **0.6**	**10** ** ± ** **0.0**	**7.7** ** ± ** **0.6**	**20.3** ** ± ** **0.6**	**17.3** ** ± ** **2.5**	**12.7** ** ± ** **1.5**	**10.7** ** ± ** **0.6**	**10** ** ± ** **0.0**	**8** ** ± ** **0.0**
**Lavender**	10 ± 0.0	9.3 ± 1.2	8 ± 1.0	7 ± 0.0	7 ± 0.0	7 ± 0.0	9.7 ± 0.6	9.3 ± 0.6	8.3 ± 1.5	7 ± 0.0	7 ± 0.0	7 ± 0.0
**Cistus**	10.3 ± 2.5	8 ± 0.0	7 ± 0.0	7 ± 0.0	7 ± 0.0	0.0 ± 0.0	10.7 ± 3.8	8 ± 0.0	7 ± 0.0	0.0 ± 0.0	0.0 ± 0.0	0.0 ± 0.0
**Helichrysum**	0.0 ± 0.0	0.0 ± 0.0	0.0 ± 0.0	0.0 ± 0.0	0.0 ± 0.0.	0.0 ± 0.0	0.0 ± 0.0	0.0 ± 0.0	0.0 ± 0.0	0.0 ± 0.0	0.0 ± 0.0	0.0 ± 0.0
**Essential Oil**	***E. coli* (*mcr*-1) **											
	**24 h**						**48 h and 72 h**					
	**50%**	**25%**	**12.5%**	**6.25%**	**3.125%**	**1.56%**	**50%**	**25%**	**12.5%**	**6.25%**	**3.125%**	**1.56%**
**Peppermint**	10.3 ± 0.6	9.3 ± 0.6	8 ± 1.0	7.7 ± 0.6	7 ± 0.0	7 ± 0.0	9.7 ± 0.6	8.7 ± 0.6	7.7 ± 0.6	7.7 ± 0.6	7 ± 0.0	7 ± 0.0
**Oregano**	**24** ** ± ** **1.0**	**22** ** ± ** **1.0**	**20.7** ** ± ** **1.2**	**17.3** ** ± ** **0.6**	**13.3** ** ± ** **0.6**	**10.3** ** ± ** **0.6**	**24.7** ** ± ** **0.6**	**21** ** ± ** **1.0**	**20** ** ± ** **0.0**	**16** ** ± ** **1.7**	**12.7** ** ± ** **0.6**	**10** ** ± ** **0.0**
**Dittany**	**22** ** ± ** **1.0**	**21** ** ± ** **1.0**	**16** ** ± ** **1.7**	**10** ** ± ** **0.0**	**9** ** ± ** **0.0**	**8.3** ** ± ** **0.6**	**21** ** ± ** **1.0**	**20.3** ** ± ** **0.6**	**13.7** ** ± ** **0.6**	**9.3** ** ± ** **0.6**	**8** ** ± ** **0.0**	**8** ** ± ** **0.0**
**Rosemary**	9 ± 0.0	8.7 ± 0.6	8 ± 0.0	7.3 ± 0.6	7 ± 0.0	0.0 ± 0.0	8.7 ± 0.6	9 ± 1.0	8.3 ± 0.6	7.3 ± 0.6	0.0 ± 0.0	0.0 ± 0.0
**Thyme**	**23.7** ** ± ** **0.6**	**19.3** ** ± ** **1.2**	**0.0** ** ± ** **0.0**	**10** ** ± ** **0.0**	**9.7** ** ± ** **0.6**	**8.3** ** ± ** **0.6**	**22.7** ** ± ** **1.5**	**15.7** ** ± ** **4.9**	**16** ** ± ** **1.7**	**10** ** ± ** **0.0**	**9.7** ** ± ** **0.6**	**8.7** ** ± ** **0.6**
**Lavender**	10 ± 0.0	9 ± 0.0	8 ± 0.0	7.3 ± 0.6	7 ± 0.0	7 ± 0.0	9.7 ± 0.6	9 ± 0.0	8 ± 0.0	7 ± 0.0	7 ± 0.0	7 ± 0.0
**Cistus**	0.0 ± 0.0	7 ± 0.0	7 ± 0.0	7 ± 0.0	0.0 ± 0.0	0.0 ± 0.0	0.0 ± 0.0	7 ± 0.0	7 ± 0.0	7 ± 0.0	0.0 ± 0.0	0.0 ± 0.0
**Helichrysum**	0.0 ± 0.0	0.0 ± 0.0	0.0 ± 0.0	0.0 ± 0.0	0.0 ± 0.0	0.0 ± 0.0	0.0 ± 0.0	0.0 ± 0.0	0.0 ± 0.0	0.0 ± 0.0	0.0 ± 0.0	0.0 ± 0.0
**Essential Oil**	***E. coli*** ** (*blaOXA*)**											
	**24 h**						**48 h and 72 h**					
	**50%**	**25%**	**12.5%**	**6.25%**	**3.125%**	**1.56%**	**50%**	**25%**	**12.5%**	**6.25%**	**3.125%**	**1.56%**
**Peppermint**	10 ± 1.0	8.3 ± 0.6	8 ± 0.0	8 ± 0.0	8 ± 0.0	7 ± 0.0	8.7 ± 0.6	7.7 ± 0.6	7.3 ± 0.6	7.3 ± 0.6	7 ± 0.0	7 ± 0.0
**Oregano**	**OMZ**	**40.3** ** ± ** **1.5**	**30.7** ** ± ** **3.8**	**18.3** ** ± ** **0.6**	**13.7** ** ± ** **0.6**	**10** ** ± ** **0.0**	**OMZ**	**38.3** ** ± ** **2.9**	**32.3** ** ± ** **2.5**	**18.3** ** ± ** **0.6**	**13.3** ** ± ** **0.6**	**10.3** ** ± ** **0.6**
**Dittany**	**35.7** ** ± ** **3.5**	**31.7** ** ± ** **1.5**	**21.3** ** ± ** **3.1**	**11.7** ** ± ** **0.6**	**9.2** ** ± 0.6**	**8.3** ** ± ** **0.6**	**35** ** ± ** **1.0**	**31.7** ** ± ** **1.5**	**19** ** ± ** **1.7**	**10.7** ** ± ** **1.2**	**9.16** ** ± 0.6**	**8.3** ** ± ** **0.6**
**Rosemary**	8.7 ± 0.6	8 ± 0.0	8 ± 0.0	8 ± 0.0	7 ± 0.0	0.0 ± 0.0	8.3 ± 0.6	8 ± 0.0	8 ± 0.0	7 ± 0.0	7 ± 0.0	0.0 ± 0.0
**Thyme**	**OMZ**	**37.7** ** ± ** **2.5**	**26.7** ** ± 0.6**	**14.7** ** ± ** **0.6**	**10.7** ** ± ** **0.6**	**9.7** ** ± ** **0.6**	**OMZ**	**35.7** ** ± ** **1.2**	**26** ** ± ** **3.6**	**13.7** ** ± ** **0.6**	**11.3** ** ± ** **0.6**	**9.3** ** ± ** **0.6**
**Lavender**	12.7 ± 1.2	10.3 ± 0.6	8.7 ± 0.6	8.3 ± 0.6	7 ± 0.0	7 ± 0.0	11 ± 0.0	8.7 ± 0.6	8 ± 0.0	8 ± 0.0	7 ± 0.0	7 ± 0.0
**Cistus**	9.7 ± 1.5	8 ± 0.0	8 ± 0.0	7.3 ± 0.6	7.3 ± 0.6	7 ± 0.0	9.3 ± 1.2	8 ± 0.0	8 ± 0.0	7 ± 0.0	7 ± 0.0	0.0 ± 0.0
**Helichrysum**	0.0 ± 0.0	8 ± 0.0	7 ± 0.0	7 ± 0.0	7 ± 0.0	7 ± 0.0	0.0 ± 0.0	7.7 ± 0.6	7.3 ± 0.6	7 ± 0.0	7 ± 0.0	7 ± 0.0
**Essential Oil**	***E. *** ***coli O157:H7***											
	**24 h**						**48 h and 72 h**					
	**50%**	**25%**	**12.5%**	**6.25%**	**3.125%**	**1.56%**	**50%**	**25%**	**12.5%**	**6.25%**	**3.125%**	**1.56%**
**Peppermint**	0.0 ± 0.0	8.7 ± 0.6	8 ± 1.0	7 ± 0.0	7 ± 0.0	7 ± 0.0	0.0 ± 0.0	8.3 ± 0.6	8 ± 1.0	7 ± 0.0	7 ± 0.0	7 ± 0.0
**Oregano**	**28** ** ± ** **1.0**	**23.3** ** ± ** **1.2**	**21** ** ± ** **1.7**	**17.3** ** ± ** **0.6**	**10.3** ** ± ** **0.6**	**10** ** ± ** **0.0**	**24.7** ** ± ** **0.6**	**21.3** ** ± ** **1.2**	**20** ** ± ** **1.0**	**16** ** ± ** **1.0**	**10** ** ± ** **0.0**	**9.7** ** ± ** **0.6**
**Dittany**	**23.7** ** ± ** **4.7**	**19** ** ± ** **1.0**	**16.3** ** ± ** **1.5**	**12.3** ** ± ** **0.6**	**9** ** ± ** **1.0**	**7.7** ** ± ** **0.6**	**22.7** ** ± ** **3.8**	**18.3** ** ± ** **1.5**	**15.3** ** ± ** **1.5**	**11** ** ± ** **1.0**	**8.7** ** ± ** **0.6**	**8** ** ± ** **0.0**
**Rosemary**	7.7 ± 0.6	8.3 ± 1.5	7.7 ± 0.6	7 ± 0.0	0.0 ± 0.0	0.0 ± 0.0	8 ± 0.0	8 ± 1.7	7.7 ± 0.6	7 ± 0.0	0.0 ± 0.0	0.0 ± 0.0
**Thyme**	**25.3** ** ± ** **1.5**	**22.3** ** ± ** **1.5**	**15.5** ** ± ** **2.1**	**13** ** ± ** **1.0**	**10** ** ± ** **0.0**	**8** ** ± ** **0.0**	**23.7** ** ± ** **1.2**	**21.7** ** ± ** **2.1**	**14.5** ** ± ** **0.7**	**12** ** ± ** **1.0**	**9.3** ** ± ** **0.6**	**8** ** ± ** **0.0**
**Lavender**	11.3 ± 0.6	9.7 ± 1.2	8.7 ± 1.2	7.7 ± 0.6	7 ± 0.0	7 ± 0.0	10.3 ± 0.6	9 ± 1.0	8.7 ± 0.6	7.7 ± 0.6	7 ± 0.0	7 ± 0.0
**Cistus**	7.5 ± 0.7	7 ± 0.0	7.3 ± 0.6	0.0 ± 0.0	0.0 ± 0.0	0.0 ± 0.0	7 ± 0.0	7 ± 0.0	7.3 ± 0.6	0.0 ± 0.0	0.0 ± 0.0	0.0 ± 0.0
**Helichrysum**	0.0 ± 0.0	7 ± 0.0	7 ± 0.0	7 ± 0.0	0.0 ± 0.0	0.0 ± 0.0	0.0 ± 0.0	7 ± 0.0	7 ± 0.0	7 ± 0.0	0.0 ± 0.0	0.0 ± 0.0

Values represent mean ± standard deviation (SD) from three independent experiments (n = 3). EO concentrations (% *v*/*v*): 50%, 25%, 12.5%, 6.25%, 3.125% and 1.56%. “OMZ” (Over Maximum Zone): inhibition zone > 50 mm.

**Table 2:** Antimicrobial susceptibility of *E. coli* strains to antibiotics.

Strain (*E. coli*)	Ampicillin		Erythromycin		Oxytetracycline	
	Zones (mm)	Interpretation	Zones (mm)	Interpretation	Zones (mm)	Interpretation
**lenticule**	**23.0**	**S**	13.0	ND	27.0	ND
***pNorm***	**14.0**	**R**	15.0	ND	34.0	ND
***mecA***	**0.0**	**R**	0.0	ND	0.0	ND
***mcr*** **-1**	**0.0**	**R**	0.0	ND	0.0	ND
***blaOXA***	**0.0**	**R**	11.0	ND	33.0	ND
**O157:H7**	**20.0**	**S**	16.0	ND	26.0	ND

Interpretation based on EUCAST v.2025 breakpoints for ampicillin only. For erythromycin and oxytetracycline, no EUCAST criteria exist for Enterobacterales. Abbreviations: **S** = Susceptible (inhibition zone ≥ breakpoint according to EUCAST v.2025). **R** = Resistant (inhibition zone < breakpoint according to EUCAST v.2025). ND = Not Defined (no official EUCAST breakpoint available for this antibiotic–strain combination).

**Table 3:** Minimum inhibitory concentration and minimum bactericidal concentration values (% *v*/*v*) of essential oils against *E. coli* strains.

EOs	*E. coli* Lenticule		*E. coli* (*pNorm*)		*E. coli* (*mecA*)		*E. coli* (*mcr*-1)		*E. coli* (*blaOXA*)		*E. coli* O157:H7	
	MIC	MBC	MIC	MBC	MIC	MBC	MIC	MBC	MIC	MBC	MIC	MBC
**Peppermint**	1%	2%	1%	2%	2%	2%	2%	2%	2%	2%	2%	2%
**Oregano**	**0.03125%**	**0.25%**	**0.0625%**	**0.25%**	**0.125%**	**0.125%**	**0.125%**	**0.125%**	**0.03125%**	**0.125%**	**0.0625%**	**0.0625%**
**Dittany**	**0.125%**	**0.5%**	**0.25%**	**0.5%**	**0.5%**	**0.5%**	**0.25%**	**0.25%**	**0.25%**	**0.25%**	**0.5%**	**0.5%**
**Rosemary**	2%	2%	2%	2%	2%	2%	2%	2%	2%	2%	2%	2%
**Thyme**	**0.5%**	**0.5%**	**0.125%**	**0.125%**	**0.125%**	**0.125%**	**0.25%**	**0.25%**	**0.25%**	**0.25%**	**0.25%**	**0.25%**
**Lavender**	2%	>2%	0.5%	1%	1%	1%	1%	1%	1%	2%	2%	2%
**Cistus**	2%	>2%	2%	>2%	2%	>2%	2%	>2%	2%	>2%	2%	>2%
**Helichrysum**	ND	ND	ND	ND	ND	ND	ND	ND	ND	ND	2%	>2%

“>2%” indicates that the bactericidal concentration exceeded the highest concentration tested (2% *v*/*v*). “ND” = Not Determined.

**Table 4:** Botanical information, plant parts used and geographical origin of the essential oils studied.

Essential Oil	Botanical Name	Plant Family	Harvest Region	Plant Part Used
Peppermint	*Mentha* × *piperita*	*Lamiaceae*	Greece	Aerial parts
Oregano	*Origanum vulgare subsp. hirtum (91.3% carvacrol)*	*Lamiaceae*	Greece	Aerial parts of flowering plant
Dittany	*Origanum dictamnus*	*Lamiaceae*	Crete, Greece	Aerial parts of flowering plant
Rosemary	*Rosmarinus officinalis*	*Lamiaceae*	Greece	Aerial parts
Thyme	*Thymus vulgaris*	*Lamiaceae*	Greece	Flowering tops
Lavender	*Lavandula angustifolia cv. Hemus* and *Lavandula angustifolia cv.*	*Lamiaceae*	Nea Tenedos, Northern Greece	Dried flowers and flowering tops
Cistus	*Cistus ladanifer*	*Cistaceae*	Nea Tenedos, Northern Greece	Leaves
Helichrysum	*Helichrysum italicum*	*Asteraceae*	Vavdos, Halkidiki, Central Macedonia	Flowering tops

**Table 5:** List of volatile compounds identified in ΕOs by GC–MS analysis and their chemical classification.

EOs	Volatile Metabolites (%)	Total (%)	Density (g/mL) 25 °C
**Peppermint**	Menthol (46), Menthone (24.7), 1.8-Cineole (6.7), Menthyl Acetate (5.4)	91	0.896
	M.H. 2.50%, O.M. 88.60%		
**Oregano**	Carvacrol (91.30)	97.29	0.95
	M.H. 2.38%, O.M. 93.44%, S.H. 0.96%, O.S. 0.44%		
**Dittany**	Carvacrol (54.81), p-Cymene (13.99), γ-Terpinene (8.33)	95.06	0.95
	M.H. 32.57%, O.M. 57.76%, S.H. 3.70%		
**Rosemary**	Camphor (26.91), α-Pinene (14.76), 1,8-Cineole (13.41), Camphene (7.31), β-Myrcene (5.15)	93.22	0.879
	M.H. 39.65%, O.M. 50.98%, S.H. 1.09%		
**Thyme**	p-Cymene (33.53), Thymol (26.59), 1,8-Cineole (6.25), Limonene (5.32)	90.59	0.9189
	M.H. 47.61%, O.M. 39.06%, S.H. 2.28%		
**Lavender**	Linalyl Acetate (31.82), Linalool (29.27), Cis-β-Ocimene (6.47), Terpinen-4-ol (5.05)	97.31%	0.885–0.905
	M.H. 11.46%, O.M. 57.76%, S.H. 6.18%		
**Cistus**	α-Pinene (38.94)	71.74	0.962
	M.H. 52.88%, O.M. 12.82%, S.H. 0.64%, O.S. 2.67%		
**Helichrysum**	γ-Curcumene (14.94), α-Pinene (14.94), Neryl acetate (14.14), β-Selinene (5.09)	79.17	0.9
	M.H. 18.23%, O.M. 19.71%, S.H. 37.30%		

Data were based on reports provided by Vessel Essential Oils’ manufacturers, with only dominant components (>5%) reported and minor ones omitted for clarity. Abbreviations: M.H.: monoterpene hydrocarbons; O.M.: oxygenated monoterpenes; S.H.: sesquiterpene hydrocarbons; O.S.: oxygenated sesquiterpenes.

## Data Availability

The data presented in this study are available on request from the corresponding author.

## Abbreviations

The following abbreviations are used in this manuscript:

AMR	Antimicrobial Resistance
EO	Essential Oil
MIC	Minimum Inhibitory Concentration
MBC	Minimum Bactericidal Concentration
CC-MS	Gass Chromatography–Mass Spectrometry
LPS	Lipopolysaccharide
GRAS	Generally Recognized As Safe
FDA	Food and Drug Administration
M.H	Monoterpene Hydrocarbons
O.M	Oxygenated Monoterpenes
S.H	Sesquiterpene Hydrocarbons
O.S	Oxygenated Sesquiterpenes
